# Cardioprotection of ischaemic preconditioning is associated with inhibition of translocation of MLKL within the plasma membrane

**DOI:** 10.1111/jcmm.13697

**Published:** 2018-06-19

**Authors:** Adrián Szobi, Veronika Farkašová‐Ledvényiová, Martin Lichý, Martina Muráriková, Slávka Čarnická, Tatiana Ravingerová, Adriana Adameová

**Affiliations:** ^1^ Faculty of Pharmacy Comenius University in Bratislava Bratislava Slovakia; ^2^ Centre of Experimental Medicine Institute for Heart Research Slovak Academy of Sciences Bratislava Slovakia

**Keywords:** heart, ischaemia‐reperfusion injury, necroptosis, RIP1 inhibition

## Abstract

Necroptosis, a form of cell loss involving the RIP1‐RIP3‐MLKL axis, has been identified in cardiac pathologies while its inhibition is cardioprotective. We investigated whether the improvement of heart function because of ischaemic preconditioning is associated with mitigation of necroptotic signaling, and these effects were compared with a pharmacological antinecroptotic approach targeting RIP1. Langendorff‐perfused rat hearts were subjected to ischaemic preconditioning with or without a RIP1 inhibitor (Nec‐1s). Necroptotic signaling and the assessment of oxidative damage and a putative involvement of CaMKII in this process were analysed in whole tissue and subcellular fractions. Ischaemic preconditioning, Nec‐1s and their combination improved postischaemic heart function recovery and reduced infarct size to a similar degree what was in line with the prevention of MLKL oligomerization and translocation to the membrane. On the other hand, membrane peroxidation and apoptosis were unchanged by either approach. Ischaemic preconditioning failed to ameliorate ischaemia–reperfusion‐induced increase in RIP1 and RIP3 while pSer229‐RIP3 levels were reduced only by Nec‐1s. In spite of the additive phosphorylation of CaMKII and PLN because of ditherapy, the postischaemic contractile force and relaxation was comparably improved in all the intervention groups while antiarrhythmic effects were observed in the ischaemic preconditioning group only. Necroptosis inhibition seems to be involved in cardioprotection of ischaemic preconditioning and is comparable but not intensified by an anti‐RIP1 agent. Changes in oxidative stress nor CaMKII signaling are unlikely to explain the beneficial effects.

## 1 INTRODUCTION

Exposure to brief periods of ischaemia and reperfusion has been recognized as a nonpharmacological approach to condition the heart to withstand a lethal dose of this stressor. This adaptation of the heart to ischaemic episodes, called myocardial ischaemic preconditioning (PC), prevents the death of cardiac cells thereby allowing for greater resistance against ischaemic (I) and reperfusion (R) arrhythmias 1 and improves recovery of contractile function.^2^ Myriad complex mechanisms involving the activation of certain triggers (metabolic products, autacoids, cytokines, neurotransmitters) promoting intracellular signalling such as the NO cascade, the reperfusion injury salvage kinase (RISK) pathway and the survival activating factor enhancement (SAFE) have been suggested to underlie the phenotypes of the conditioned heart.^3^ Although they can promote survival pathways and thereby mediate PC‐induced cardiac salvage, the role of particular types of cell death is less understood and the importance of individual mechanisms is still a subject of investigation. As a consequence of PC, apoptosis and passive necrosis have been shown to occur at a lower extent 4, 5 and autophagy stimulation during the preconditioning period has been suggested to increase resistance against ischaemia/reperfusion (I/R) injury.^6^, ^7^

In recent few years, a form of cell death resembling necrotic phenotype and being dependent on RIP1‐RIP3‐MLKL signaling has been identified in ischaemic/reperfused hearts.^8^, ^9^, ^10^, ^11^ Likewise, this type of cell loss has been proven in postmyocardial infarction remodelling and heart failure and hypothesized to account, at least in part, for cardiac dysfunction and disease progression.^11^, ^12^, ^13^ Unfortunately, very little is known about mechanisms of necroptosis induction which sensitize cardiac cells to die because of I/R or other stressors. On the other hand, necroptosis propagation and execution have been studied in detail throughout recent years. Necroptosis might involve the formation of a large amyloid‐like cellular structure called the necrosome 14 which is followed by disruption of the cellular membrane as a result of translocation of MLKL, a terminal necroptotic protein, into the plasma membrane.^15^, ^16^ Conversely, inhibition of necroptotic pathway targeting RIP1/RIP3 has been shown to prevent necrosome formation and necroptotic cell death.^17^ In the context of I/R injury, control of necroptotic cell death by RIP1 inhibition has been shown to ameliorate contractile function, and prevent further adverse remodelling because of acute I/R.^9^, ^10^, ^18^

In view of the fact that PC is cardioprotective, we tested the hypothesis that its beneficial effects can be attributed to preserved cardiac cell viability because of the limitation of necroptotic cell death. Furthermore, we hypothesized that a simultaneously applied cardioprotective approach involving necroptosis inhibition (by targeting RIP1) and PC possess a greater benefit than either of the interventions applied solely. In addition, because CaMKII has been linked with both PC and RIP3 signaling,^13^, ^19^, ^20^ we assessed its contribution to cardioprotection. Lastly, because increased reactive oxygen species (ROS) production has been associated with necroptosis induction and execution^17^, ^21^, ^22^ and is consistently associated with detrimental postischaemic phenotypes^23^ we also investigated a possible mechanistic role of oxidative stress.

## 2 MATERIALS AND METHODS

### 2.1 Animals and experimental groups

Protocol of the study has been approved by the Ethics Committee of the Faculty of Pharmacy, Comenius University. All described procedures were performed in accordance with the Guide for the care and Use of Laboratory Animals, published by the US National Institutes of Health (NIH publication No 85‐23, revised in 1996), preceding an authorization by the Animal Care and Use Committee of Slovak Republic.

Male Wistar rats (weight: 200‐250 g; 14‐16 weeks old) were housed under standard conditions with a constant 12:12‐hours light/dark cycle (lights on 06:00) and a temperature of 22 ± 2°C. Animals were fed a standard pelleted diet with ad libitum water intake.

**Figure 1:**
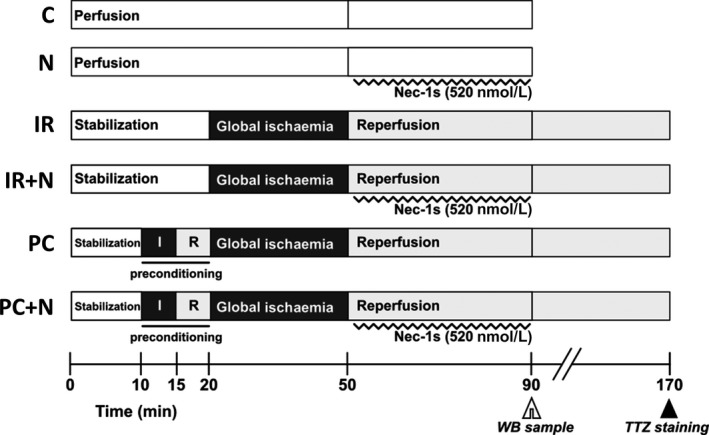
An illustration depicting used perfusion‐only, ischaemia/reperfusion or ischaemic preconditioning protocols to investigate signaling pathways and mechanical function of the heart and infarct size C—nonischaemia control; N—nonischaemic group treated with Nec‐1s; IR—I/R group; PC—group subjected to a single 5‐min preconditioning cycle prior to I/R; IR+N—I/R group treated with Nec‐1s during reperfusion; PC+N—group subjected to preconditioning and treated with Nec‐1s

After adaptation, animals were randomized into 6 groups to be further subjected to perfusion‐only (control), ischaemia/reperfusion (I/R) or ischaemic preconditioning (PC) protocols. Each group was treated either with a vehicle (0.004% v/v DMSO) or with Nec‐1s, a RIP1 inhibitor,^24^ which was present in the perfusion buffer at a concentration of 520 nmol/L during the first of 40‐minutes perfusion/reperfusion, giving 6 individual groups (Figure 1).

### 2.2 Experimental I/R protocol

Rats were anaesthetized by administration of sodium pentobarbital (60 mg/kg, ip) with heparin (1000 IU/kg, ip), and hearts were quickly removed and retrograde perfused in Langendorff mode. Perfusion was performed with a constant perfusion pressure of 70 mm Hg and a temperature of 37.5 ± 0.2°C. Perfusion solution was a modified Krebs‐Henseleit buffer with pH = 7,4, gassed with 95% O_2_ and 5% CO_2_ and with the following composition: 118 mmol/L NaCl; 3.2 mmol/L KCl; 1.2 mmol/L MgSO_4_; 25 mmol/L NaHCO_3_; 1.18 mmol/L KH_2_PO_4_; 1.25 mmol/L CaCl_2_; 11 mmol/L glucose.

After time‐matched aerobic perfusion during which measured functional parameters were constant, a 30‐minute‐long global ischaemia was induced by stopping the perfusion pump, followed by a 40‐minute reperfusion. In preconditioned groups, prior to I/R protocol, ischaemic preconditioning was induced by a short 5‐minute‐long period of global ischaemia followed by 5 minutes of reperfusion. Throughout the protocol, ECG and mechanical function of the heart were monitored. After the end of the protocol, the hearts were frozen for biochemical and molecular studies. Separate series of hearts from the vehicle‐, preconditioning‐ and Nec‐1s‐treated ischaemic hearts were subjected to 120‐minute reperfusion to measure infarct size with triphenyltetrazolium chloride staining.

Left ventricular developed pressure (LVDP), left ventricular end‐diastolic pressure (LVEDP) and indices of contraction and relaxation +(d*P*/d*t*) and −(d*P*/d*t*) were measured with PowerLab/8SP Chart 7 software. LVDP, ±(d*P*/d*t*)max are expressed as percentage of baseline (end of stabilization) values and in case of LVEDP as difference from baseline to control for inherent mechanical parameter variability. Epicardial electrocardiogram was recorded using 2 electrodes connected to the apex of the heart and the aorta. Severity of arrhythmias was analysed using guidelines of The Lambeth Conventions.^25^

### 2.3 Assessment of infarct size with triphenyltetrazolium chloride (TTZ) staining

Hearts subjected to I/R reperfused for 120 minutes were perfused with a 1% (w/v) TTZ solution for 2 minutes and were thereafter left to incubate in the same solution for 30 minutes at 37°C. Then, the hearts were fixed in 10% (v/v) formaldehyde for 24 hours, cut to 1‐mm‐thick slices and incubated in 10% (v/v) formaldehyde once again. Infarct size was analysed in a blinded manner by planimetric software. Infarct size was expressed as a fraction of area at risk which represents both infarcted and surviving tissue.

### 2.4 Subcellular fractionation protocol

Subcellular fractionation was performed according to 2 previously published methods 26, 27 with some modifications. At all times, samples were kept at 4°C or lower and all buffer solutions contained phosphatase and protease inhibitors (1 mmol/L PMSF, 2.5 μg/μL leupeptin, 2 μg/μL pepstatin A, 15 mmol/L NaF, 1 mmol/L Na_3_VO_4_). Left ventricular tissue samples (~30 mg) were first homogenized in liquid nitrogen with a base buffer and were left on ice for 30 minutes. Afterwards, successive centrifugation steps were performed to sequentially isolate the cytosolic, membrane and nuclear fractions. Details of this procedure can be found in the online‐only supplementary material.

### 2.5 SDS‐PAGE and immunoblotting

Total left ventricular samples were prepared by homogenizing tissue in liquid nitrogen with a modified RIPA buffer (40 mmol/L Tris‐HCl, pH = 7,6; 100 mmol/L NaCl; 0.5 mmol/L EDTA; 1 mmol/L EGTA; 1% v/v; Triton X‐100; 0.5% w/v Na‐deoxycholate; 1% w/v SDS; 5% v/v glycerol) containing protease (1 mmol/L PMSF; 2.5 μg/mL leupeptin; 2 μg/mL pepstatin A) and phosphatase (15 mmol/L NaF; 1 mmol/L Na_3_VO_4_) inhibitors, and concentration of proteins in prepared samples was determined by Lowry assay. Proteins (35 μg/sample for total lysates; 8 μg/sample for fractions) were separated electrophoretically by standard reducing or nonreducing SDS‐PAGE (hand‐cast 8%, 10% or 12% Bis‐tris/MOPS gels) and transferred on PVDF (polyvinylidene difluoride) membranes (Immobilon P, Millipore, USA). Membranes were blocked with 2% BSA in TBST and incubated with primary antibodies against proteins of interest followed by secondary antibodies. Details about used antibodies can be found in the online‐only supplement. Signal was detected by the combination of a chemiluminescence kit (Luminata, Millipore, USA) and myECL Imager (Thermo Fischer Scientific, USA) and analysed with myImage Analysis software (Thermo Fischer Scientific). All signals were normalized to total protein staining with Ponceau S (total lysates) or Copper(II) phthalocyanine‐tetrasulfonate (subcellular fractions; higher sensitivity) to control for the amount of loaded protein.^28^

### 2.6 Lipoperoxidation measurement by thiobarbituric acid reactive substances (TBARS) assay

As a marker of oxidative stress and lipoperoxidation, TBARS level in total left ventricular tissue lysates was analysed by the method according to Shlafer and Shepard 29 with modifications. In short, a 1:1 mixture of tissue homogenates and 20% (w/v) trichloroacetic acid solution was mixed with a fourfold excess of TBARS reagent (37 mmol/L thiobarbituric acid, 500 mmol/L NaOH, 15% v/v acetic acid) and heated to 95°C for 70 minutes. Afterwards, the mixture was left to cool and the pink product was extracted into a 15:1 mixture of 1‐butanol/pyridine 535 nm absorbance of which was read by a spectrophotometer (ELx800, BioTek, USA). All analyses were performed in duplicate, and their averages were used to calculate the concentration of malondialdehyde (MDA) equivalents in samples according to a calibration curve made from tetrabutylammonium malondialdehyde salt. The final concentration of MDA equivalents in samples was normalized to protein concentration as determined by Lowry assay.

### 2.7 Materials

All materials with the exception of antibodies used in the study were obtained from one of the following companies: Sigma‐Aldrich (DE), Merck (DE), CentralChem (SK), Abcam (UK), Apollo Scientific (UK).

### 2.8 Statistical analysis

Data are expressed as the mean ± SEM for the number (n) of animals in the group. Two‐way ANOVA (2WA) and Bonferroni's test were applied for comparison of differences in variables with normal distribution between the 4 groups exposed to I/R (factor “N”—presence/absence of Nec‐1s; factor “PC”—presence/absence of PC). Because of non‐normality, average ventricular tachycardia durations were first log10‐transformed before being subjected to 2WA. Mixed‐model ANOVA (MMA) was used to compare time‐course data of I/R groups (factor “*t*”—time; factor “grp”—group). Incidence of arrhythmias was evaluated with chi‐squared test followed by pairwise chi‐squared tests with Bonferroni's correction to compare individual pairs of groups. Kruskal‐Wallis test and post hoc Dunn's test were used to analyse arrhythmia score. Two‐tailed Student's *t* test (with or without Welch's correction) was applied to comparisons of normally distributed data between only 2 groups. Statistical significance of changed membrane MLKL levels was tested by Fisher's exact test by comparing the presence/absence of MLKL in the IR group to the other groups combined. All significant differences (*P* < 0.05) are indicated in figures.

## 3 RESULTS

### 3.1 The effects of ischaemic preconditioning, Nec‐1s and their combination on necroptosis and apoptosis

**Figure 2:**
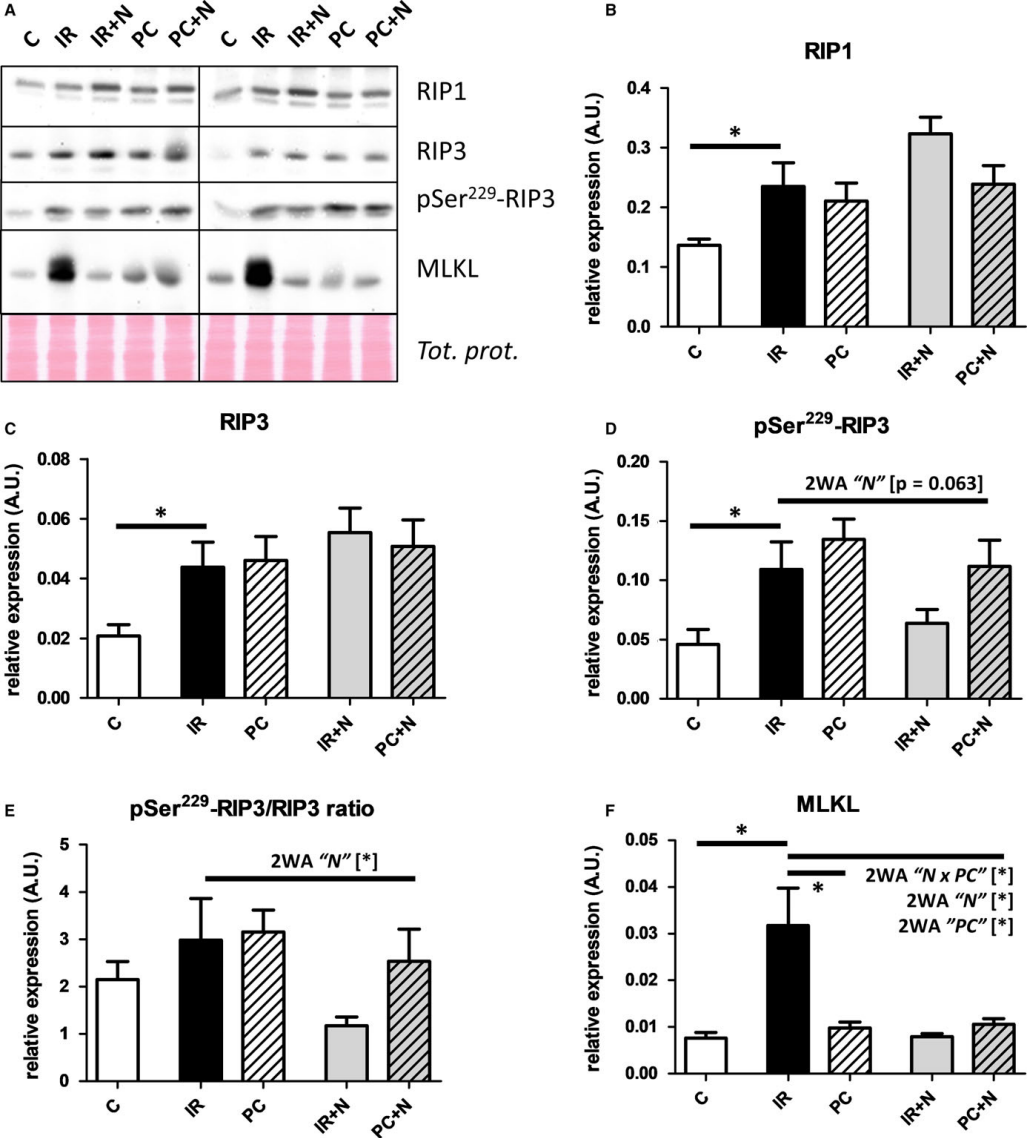
Analysis of effects of IPC and Nec‐1s on whole tissue necroptotic signaling in I/R‐damaged hearts. A, Representative immunoblots and B‐F, Immunoblot quantification of RIP1, RIP3, pSer229‐RIP3 and MLKL in perfused‐only (C) and I/R‐subjected hearts treated with vehicle (IR), IPC (PC), Nec‐1s (IR+N) and their combination (PC+N). Data are presented as mean ± SEM, n = 6/group. *P < 0.05. 2WA—2‐way ANOVA; “N” factor—presence of Nec‐1s; “PC” factor—presence of ischaemic preconditioning; “N × PC” interaction of the 2 factors

Necroptosis‐triggering kinase RIP1 was significantly elevated in all I/R groups with no apparent changes induced by PC. The levels of this protein as a target of inhibition in Nec‐1s perfused hearts were also not influenced by Nec‐1s treatment irrespective of I/R or PC protocol (Figure 2B). However, Nec‐1s perfusion by itself increased RIP1 kinase expression (Figure S2B). Likewise, analysis of RIP3 kinase expression, which is a substrate of RIP1 and can promote necroptosis, gave a result similar to that of RIP1 (Figure 2C).

In support, levels of pSer229‐RIP3 were elevated in left ventricles exposed to I/R injury and no changes in RIP3 phosphorylation were detected in PC hearts (Figure 2D). On the other hand, as could be expected based on the mechanism of action of the pharmacological intervention applied, relative Ser229‐RIP3 phosphorylation was decreased in I/R hearts subjected to Nec‐1s perfusion (Figure 2E). However, a surprising very profound increase in RIP3 Ser229 phosphorylation was observed in nonischaemic Nec‐1s‐perfused hearts (Figure S2B).

**Figure 3:**
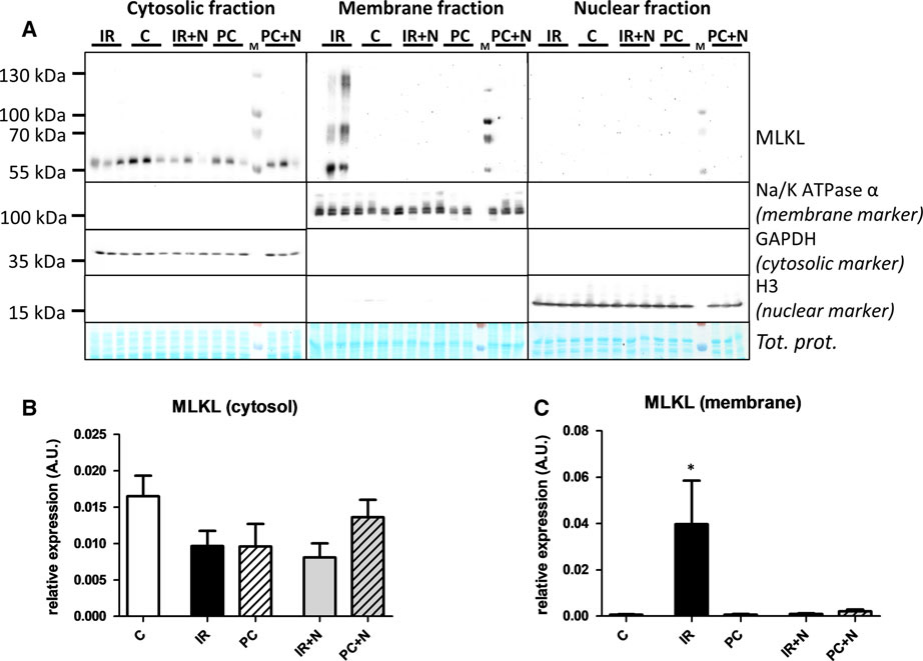
Analysis of effects of IPC and Nec‐1s on MLKL subcellular translocation under nonreducing conditions in I/R‐damaged hearts. A, Representative immunoblots of MLKL expression in cytosol, cytoplasmic membrane and nucleus (upper panel) and respective blots demonstrating the purity of particular fractions assessed by the presence of GAPDH, Na/K‐ATPase α and Histone H3 (down panel) in perfused‐only (C) and I/R‐subjected hearts treated with vehicle (IR), IPC (PC), Nec‐1s (IR+N) and their combination (PC+N) B and C, Immunoblot quantification of MLKL in cytosol and cytoplasmic membrane. Data are presented as mean ± SEM, n = 6/group. M—molecular weight marker. *P < 0.05

Importantly, MLKL, a downstream protein of RIP3 serving as the terminal effector of necroptosis, was modulated by PC, and additional treatment with Nec‐1s did not change these levels (Figure 2F). Moreover, although a comparable amount of MLKL was detected in the cytoplasm of all tested groups including the nonischaemic one (Figure 3B), plasma membrane‐associated MLKL was evidently present only in the untreated hearts subjected to I/R (Figure 3C). In contrast to the I/R group, no signal for membrane MLKL was detected in PC and Nec‐1s‐treated groups thus indicating inhibition of translocation of this protein from the cytoplasm to the membrane. Application of Nec‐1s to the perfusion medium of PC hearts did not enhance or interfere with this cardioprotection. Furthermore, MLKL in the cytoplasmic fraction of all groups was in the monomeric form, while under nonreducing conditions higher‐molecular weight forms of MLKL above the 55 kD monomeric form detected only in the membrane fraction in the untreated I/R group have indicated the presence of cytotoxic MLKL oligomers. Thus, these data suggest that PC, like Nec‐1s, inhibits necroptotic cell death by inhibition of formation of MLKL oligomers and their subsequent translocation within the plasma membrane.

**Figure 4:**
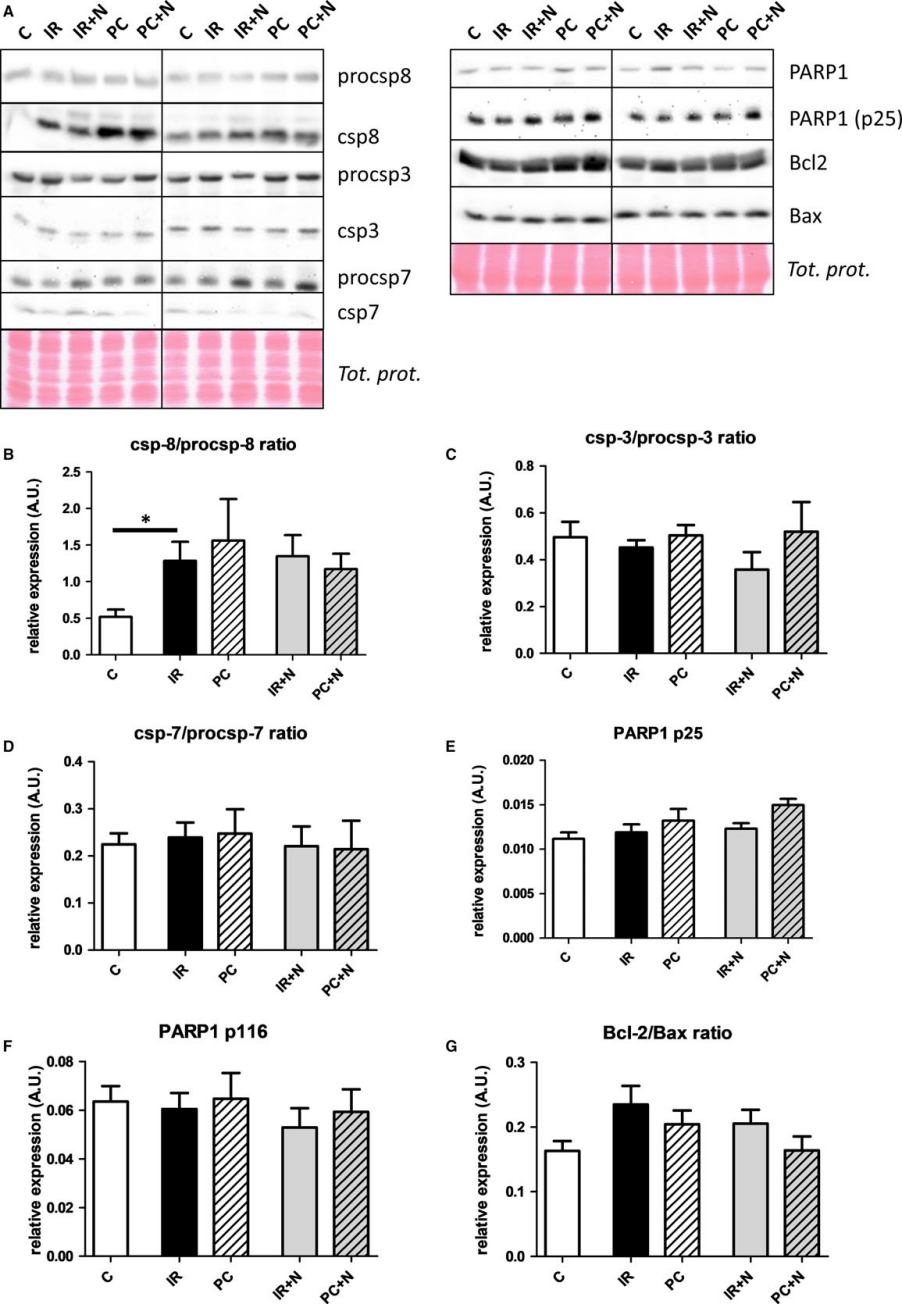
Analysis of effects of IPC and Nec‐1s on whole tissue apoptotic signaling in I/R‐damaged hearts. A, Representative immunoblots and quantification of procsp‐8/csp‐8 (B), procsp‐3/csp‐3 (C), procsp‐7/csp‐7 ratio (D), p25 PARP1 (E), p116 PARP1 (F) and Bcl‐2/Bax ratio (G) in perfused‐only (C) and I/R‐subjected hearts treated with vehicle (IR), IPC (PC), Nec‐1s (IR+N) and their combination (PC+N). Data are presented as mean ± SEM, n = 6/group. *P < 0.05

To provide more insights on regulated cell death, in particular apoptosis which counterbalances necroptosis, the expression of some apoptotic markers was analysed (Figure 4A‐G). The expression of the active p18 form of caspase‐8, known to inactivate RIP1 and RIP3 in addition to being an initiator apoptotic caspase,^30^, ^31^ was increased in all groups exposed to I/R regardless of the presence or absence of any intervention. Likewise, as the expression of the zymogenic caspase‐8 was unchanged in all groups, csp‐8/procsp‐8 ratio mirrored the expression of active csp‐8 (Figure 4B). Although these changes suggest the activation of the extrinsic pathways of apoptosis in I/R hearts, other apoptotic markers did not differ among all groups and did not indicate the involvement of apoptosis in tissue damage. Indeed, procaspase‐3, a direct substrate of caspase‐8,^32^ was not found to be cleaved more in groups with higher caspase‐8 expression (Figure 4C). Comparable results could be seen in case of another execution caspase, caspase‐7 (Figure 4D). Similarly, PARP1 cleavage, a downstream of activated executioner caspases,^33^ was in line with caspase‐3 and caspase‐7 expression data. Total PARP1 expression was constant, and the apoptosis‐specific p25 fragment (the counterpart to p89) characteristic of ongoing apoptosis was completely unaffected by I/R and PC did not change these levels (Figure 4E,F). In accordance, the final marker investigated, the ratio of antiapoptotic Bcl‐2 and proapoptotic Bax, considered to be an apoptosis susceptibility marker, also did not differ among the groups (Figure 4G). No effects of Nec‐1s on the expression of apoptotic markers were observed in nonischaemic tissues with the exception of csp‐7 which was decreased (Figure S2C,D).

### 3.2 The effects of ischaemic preconditioning, Nec‐1s and their combination on infarct size, mechanical and electrical activity of the heart

**Figure 5:**
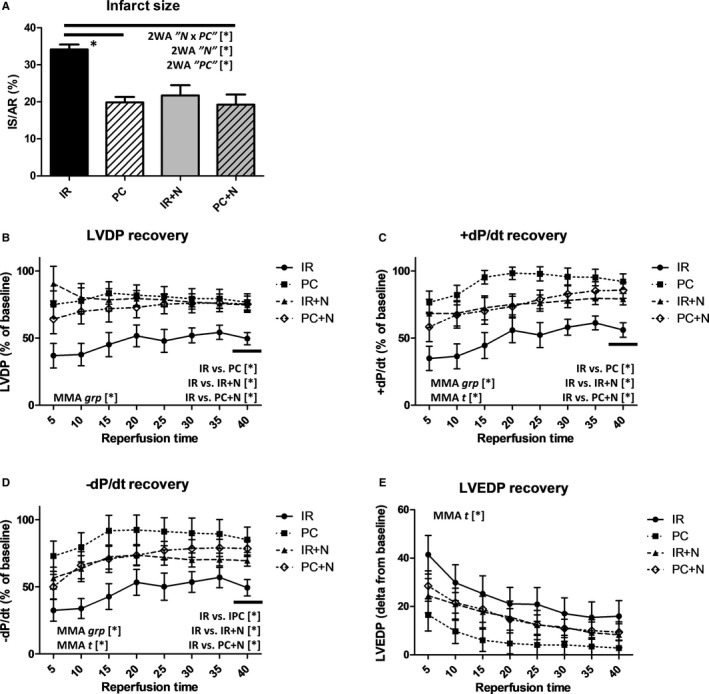
A, Analysis of effects of IPC and Nec‐1s on infarct size measured by TTZ staining of I/R‐damaged hearts treated with vehicle (IR), IPC (PC), Nec‐1s (IR+N) and their combination (PC+N). Data are presented as mean ± SEM, n = 6, 7, 9 and 9/group respectively. *P < .05. 2WA—2‐way ANOVA; “N” factor—presence of Nec‐1s; “PC” factor—presence of ischaemic preconditioning; “N × PC” interaction of the 2 factors. B‐D, Recovery of haemodynamic parameters of I/R‐damaged hearts treated with vehicle (IR), IPC (PC), Nec‐1s (IR+N) and their combination (PC+N). B) LVDP—left ventricular developed pressure, C and D, (d*P*/d*t*)max—maximal rate of contraction, −(d*P*/d*t*)max—maximal rate of relaxation, E, LVEDP—left ventricular end‐diastolic pressure. Data are presented as mean ± SEM, n = 10, 13, 15 and 15/group, respectively. *P < 0.05. MMA, Mixed‐model ANOVA; “*t*” factor—time; “grp” factor—group

The analysis of the extent of myocardial necrosis assessed as ratio of TTZ‐positive tissue area to total tissue area showed comparable antinecrotic effects of both PC and Nec‐1s (Figure 5A). Comparing the infarct sizes between groups further showed that PC did not have an additive effect on myocardial protection provided by Nec‐1s. These observations agree with the molecular data and indicate that these 2 different interventions do not interfere with each other and thus could act on the same cell death pathways.

LV pressure recordings during I/R revealed that both PC and Nec‐1s, the selective inhibitor of the necroptosis‐initiating protein RIP1, improved postischaemic restoration of myocardial contractile function post‐I/R injury during the entire R period (Figure 5B‐E). After 40 minutes of R, in all intervention groups, the recovery of LVDP was indistinguishable and significantly higher as compared to the untreated I/R group (Figure 5B). Likewise, I/R groups exposed to simultaneous PC and/or Nec‐1s treatment presented the same final LVDP recovery. The same pattern of results was also observed when comparing the contraction and relaxation indices (±d*P*/d*t*), suggesting comparable improvements in systolic and diastolic function in all treated hearts irrespective of the intervention applied (Figure 5C,D). On the other hand, a trend towards rapid recovery of the rate of relaxation and contraction was observed in the PC‐only group compared to the Nec‐1s pretreated I/R group. Contrarily to −d*P*/d*t*, 40‐minute LVEDP recovery was very heterogenous in treated groups and no significant improvement was found compared to I/R hearts; however, the PC‐only group did show a trend towards LVEDP reduction (Figure 5D).

**Figure 6:**
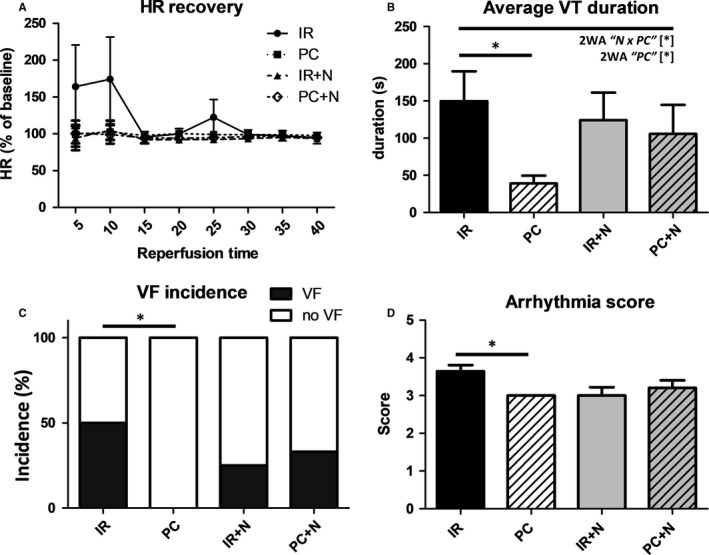
Effects of IPC and Nec‐1s on electrical activity of I/R‐damaged hearts. A, HR recovery and arrhythmias analysis assessed by B, VT duration, C, VF incidence and D, Arrhythmia score in I/R‐damaged hearts treated with vehicle (IR), IPC (PC), Nec‐1s (IR+N) and their combination (PC+N). Nonincidence data are presented as mean ± SEM, n = 10, 13, 15 and 15/group, respectively. *P < 0.05. 2WA—2‐way ANOVA; “N” factor—presence of Nec‐1s; “PC” factor—presence of ischaemic preconditioning; “N × PC” interaction of the 2 factors

Compared to preischaemic values, HR did not differ between groups at any time‐point (Figure 6A). Thus, values of rate pressure product were mostly following the pattern of LVDP (data not shown). A significant difference in the incidence and the mean duration of reperfusion arrhythmias was observed among groups (Figure 6B‐D). Well‐known cardioprotective effects of PC on arrhythmias were recapitulated in our study, and they were evidenced as significant reduction in the duration of ventricular tachycardia episodes (VT) (Figure 6B) and the incidence of ventricular fibrillation (VF) (Figure 6C). However, the intervention used to inhibit necroptosis failed to elicit this type of cardioprotection. In fact, Nec‐1s did not influence the incidence of VFs or the cumulative duration of VTs. The same pattern of arrhythmias occurrence was observed when this drug was applied together with PC suggesting an interference of necroptosis inhibition with antiarrhythmic mechanisms of PC. In support, the assessment of severity of total arrhythmogenesis reported as arrhythmia score showed significance solely for the PC group (Figure 6D).

### 3.3 The effects of ischaemic preconditioning and/or necroptosis inhibition on CaMKII‐associated signalling

**Figure 7:**
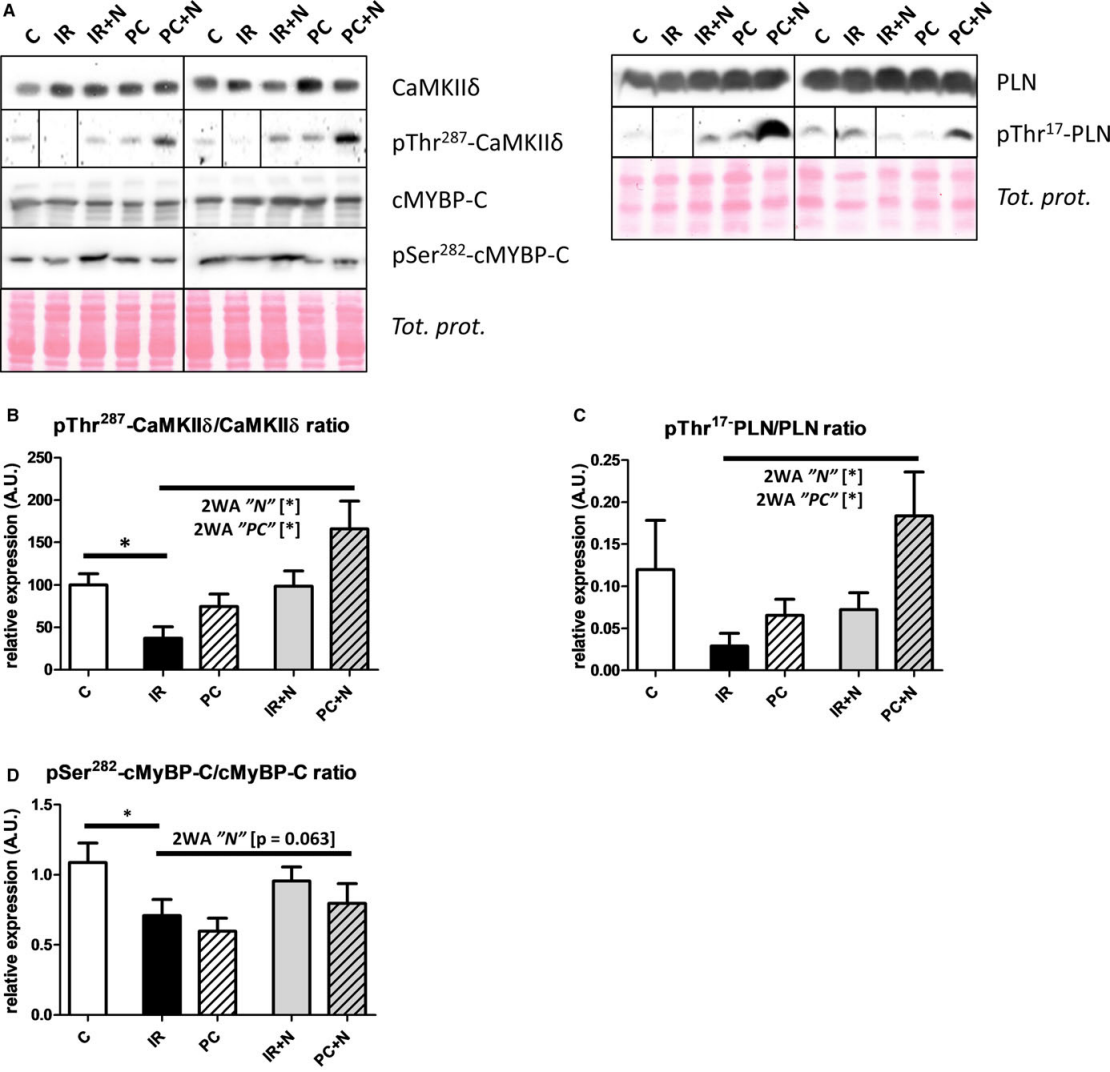
Influence of IPC and Nec‐1s on the levels and phosphorylation of CaMKIIδ, PLN and cMyBP‐C in whole tissue of I/R‐damaged hearts. A, Representative immunoblots and B‐D, quantification of ratio of pThr287‐CaMKIIδ/CaMKIIδ, pThr17‐PLN/PLN, pSer282‐cMyBP‐C/cMyBP‐C in perfused‐only (C) and I/R‐subjected hearts treated with vehicle (IR) or IPC (PC) or Nec‐1s (IR+N) or combination treatment (PC+N). Blots for pThr287‐CaMKIIδ and pThr17‐PLN are spliced because of a different loading order. Data are presented as mean ± SEM, n = 6/group. *P < 0.05. 2WA—2‐way ANOVA; “N” factor—presence of Nec‐1s; “PC” factor—presence of ischaemic preconditioning; “N × PC” interaction of the 2 factors

To provide more details on the antinecro(pto)tic effects of both interventions and possible explanations for divergences in the modulation of contractile recovery and arrhythmias, we searched for such a molecule which could be an interlink between these cellular events. To meet these criteria, we chose CaMKIIδ. The expression of total CaMKIIδ was changed by neither PC nor Nec‐1s (Figure 7B) being applied alone or together. However, the degree of phosphorylation at Thr287, the autophosphorylation site indicative of kinase activation, was affected (Figure 7B). While at the 40‐min R, the relative phosphorylation of this kinase was depressed compared to control perfused‐only hearts, PC augmented the phosphorylation of CaMKIIδ during reperfusion and independently of it Nec‐1s did the same. Indeed, pThr287‐CaMKIIδ expression was the greatest in the PC+N group. Intriguingly, in the absence of I/R damage (Figure S2E), Nec‐1s perfusion increased CaMKIIδ phosphorylation at Thr287 suggesting that RIP1 kinase may modulate the activity of this protein kinase even under baseline conditions.^34^

In line with the activation of CaMKIIδ, phosphorylation of PLN at Thr17, a downstream protein of this kinase, was found to be altered in a similar fashion (Figure 7C). Indeed, a trend for I/R‐induced drop in phosphorylation of PLN was observed while Nec‐1s and PC were capable of raising it, and this effect was additive in PC+N group. In addition, PLN phosphorylation at Thr17 also followed pThr287‐CaMKIIδ in nonischaemic, Nec‐1s perfused‐only group (Figure S2E).

In addition, the phosphorylation of the protein cMyBP‐C at Ser282 being also phosphorylated mainly by CaMKII action was also found to be decreased by I/R (Figure 7D) supporting the findings from physiological investigation. Two‐way ANOVA analysis of the 4 ischaemic groups revealed no significant changes even though there was a trend for Nec‐1s to increase the degree of Ser282‐cMyBP‐C phosphorylation. Furthermore, just like with pThr287‐CaMKIIδ, Nec‐1s perfusion of nonischaemic hearts greatly enhanced the phosphorylation at Ser282‐cMyBP‐C (Figure S2E) which, however, did not actually impact real mechanical function of the heart (data not shown).

### 3.4 The influence of ischaemic preconditioning and/or necroptosis inhibition on oxidative stress

While predictably I/R led to a significant increase in the amount of TBARS, no intervention used either alone or in combination prevented the observed rise (Figure S1). Intriguingly, perfusion of nonischaemic hearts with Nec‐1s showed a tendency towards an increase in the amount of TBARS observed (Figure S2F).

## 4 DISCUSSION

Although a vast number of studies have been carried out to examine mechanisms underlying PC‐associated mitigation of cardiac damage, this is the first study investigating its cardioprotective action with respect to modification of necroptotic cell death. We have shown that in spite of lowering infarct size, PC affects neither the total expression of main necroptotic proteins (RIP1, RIP3, MLKL) nor post‐translational modification of RIP3 (pSer229‐RIP3), an upstream activator of cytotoxic MLKL. Moreover, we have also demonstrated for the first time that PC reduces MLKL translocation into the plasma membrane indicating the retardation of necroptotic cell death which might underlie its beneficial effects. In addition, this study indicates that a combined approach involving RIP1 inhibition and PC possess neither additive nor antagonistic action.

### 4.1 PC possesses cardioprotection by retardation of necroptosis without any effects on apoptosis

Loss of viable cardiac cells is essential for determining heart function. Accordingly, increased rates of apoptotic and necrotic cell death are a constant finding in I/R hearts while PC has been found to partially mitigate the injury by various mechanisms including those interfering with cell death pathways such as caspase inhibition, TNFα depletion or specific miRNAs.^35^, ^36^, ^37^ By approach used in this study, we have provided additional insight into the mechanisms limiting cell loss by adaptation to short episodes of ischaemia. Indeed, I/R hearts expressed a higher amount of RIP1 and RIP3 consistent with previous studies^8^, ^9^, ^10^, ^38^ while neither PC nor Nec‐1s did normalize these changes. On the other hand, Nec‐1, a chemical analogue of Nec‐1s, was shown to reduce both RIP1 and RIP3 in other types of myocardial I/R models.^9^, ^10^, ^39^ RIP1 and RIP3 are important constituents of cell death mediating platforms such as TNFR1‐associated complex I and II 40 and can be inactivated by csp‐8.^30^, ^31^ In I/R‐subjected hearts, csp‐8 was found to be up‐regulated indicating the activation of extrinsic arm of the apoptotic pathway rather than promotion of necroptotic one. However, no single apoptotic marker (either the downstream proteins csp3, csp7, p25 PARP1 or proteins of the Bcl‐2 family) being changed in our samples does not support this assumption. On the other hand, it cannot be ruled out that csp‐8 participated in modulation of various noncell death‐related processes.^41^ Thus, PC was unlikely to limit cardiac damage as a result of prevention of apoptosis. This finding is in accordance with a previous study where PC was found not to interfere with apoptosis this early in reperfusion.^42^ It should be mentioned that contrary to our results, many studies investigating apoptosis, although later during reperfusion, have shown marked activation of apoptosis during I/R.^4^, ^43^, ^44^, ^45^ This difference could be because of apoptosis lagging behind necrosis during reperfusion.^42^, ^46^

In line with up‐regulation of RIP1 and RIP3, pSer229‐RIP3 expression was also higher after I/R. As a result of activating Ser229‐RIP3 phosphorylation leading into phosphorylation of MLKL at Thr357/Ser358 residues necroptosis is promoted by the formation of an oligomeric complex and its translocation to the membrane.^14^, ^15^, ^16^ Currently, we are unable to provide evidence about phosphorylation of MLKL because no commercially available antibody is able to detect this post‐translational modification of this protein in rats. However, by using subcellular fractionation we have found that PC decreased MLKL translocation into the membrane. Reduction of MLKL oligomerization because of PC indicates retardation of necroptotic cell death and thereby greater salvage of I/R hearts. These effects were accompanied by no changes in oxidative damage. Adding Nec‐1s, a RIP1 inhibitor, in the perfusion media of PC hearts did not interfere with the antinecroptotic action of PC while this agent, as could be expected, decreased RIP3 phosphorylation and also membrane MLKL levels exactly like PC. In a similar vein to necroptosis signalling, infarct size was comparably lower in groups treated with PC with or without Nec‐1s. Likewise, we observed a comparable recovery of heart function in groups subjected to these nonpharmacological and pharmacological interventions irrespective of their standalone or combined application. In contrast, only PC applied by itself mitigated arrhythmia severity.

### 4.2 Possible mechanisms of antinecroptotic effects of PC

Although PC and Nec‐1s comparably reduced IR injury, particular mechanisms of these interventions seem to be different in some points. Both interventions failed to modulate RIP1 and RIP3 expression but reduced MLKL expression in the membrane fraction. On the other hand, Nec‐1s, unlike PC, reduced pSer229‐RIP3, known to promote MLKL phosphorylation and oligomerization. Based on this, it can be postulated that while Nec‐1s likely modulates MLKL via its upstream, PC acts directly on MLKL or modulates some processes regulating MLKL phosphorylation/oligomerization independently of RIP1 or RIP3 expression and action. Moreover, it can also be highlighted that although these 2 mechanisms on MLKL translocation are likely to be different in these interventions, they affected each other neither negatively nor positively.

Although we have not designed the study to investigate these mechanisms in detail, analysis of CaMKII can provide some explanation. First, this protein kinase has been suggested to be a downstream of RIP3 and implicated in necroptosis through cyclophilin D (CypD)‐sensitive mitochondrial permeability transition pore (MPTP) induced mitochondrial depolarization both in I/R and doxorubicin‐treated rats 13 as well as in an oxygen/glucose deprivation model in oligodendrocytes.^47^ Secondly, CaMKII has been reported to induce apoptosis by promoting MPTP opening through increasing mitochondrial Ca^2+^ entry.^48^ Thus, MPTP, long understood to play a crucial role in I/R as well as in PC‐induced cardioprotection,^49^ could be an important molecular target of interventions causing necroptosis retardation. Indeed, it has been shown that cardioprotective effects of Nec‐1, the first described RIP1 inhibitor, are dependent on the presence of CypD as its inhibition by cyclosporin A 18 or its genetic ablation 50 was able to abolish its protection. On the other hand, a different study on a myocardial I/R model 10 showed that necroptosis inhibition by Nec‐1 did prevent the formation of the canonical RIP1‐RIP3‐MLKL necrosome complex during reperfusion which should be independent of MPTP. This would be in line with our observation of MLKL oligomerization and translocation to the cytoplasmic membrane in I/R‐damaged hearts. As these 2 processes, RIP1‐RIP3‐MLKL signalling and MPTP, have been observed to also occur sequentially,^51^ it is possible that such interplay between them may also play a role in I/R conditions. Analogously, as PC was shown to inhibit MPTP opening 49 and on a molecular level it prevented membrane MLKL translocation and thereby mimicked direct RIP1 inhibition to a large degree, these mechanisms, whether alone or interlinked, could also underlie the cardioprotective action of PC.

Reconsidering the role of CaMKII in these settings, we found decreased levels of CaMKIIδ activating autophosphorylation at Thr287 in I/R hearts what was prevented by PC as well as Nec‐1s administration. Interestingly, this effect was additive when the 2 approaches were combined. In accordance, pThr17‐PLN, a direct CaMKII downstream, mirrored the levels of pCaMKIIδ in the particular groups. On the one hand, these findings do not seem to fully support the aforementioned necroptosis‐CaMKII‐MPTP link.^13^, ^47^ In fact, overactivation of CaMKII 48, 52 is believed to be deleterious rather than cardioprotective. On the other, Osada, et al^19^ have suggested that such an increase in CaMKII‐mediated phosphorylation of its main SR downstream proteins (including PLN) after ischaemia may underlie beneficial effects of PC on contractile dysfunction. The normalization of I/R‐depressed phosphorylation of CaMKII and PLN could, at least in part, explain the mitigation of myocardial stunning in PC hearts and probably also in Nec‐1s‐treated hearts. In contrast, in spite of the fact that the combined group exhibited higher levels of both pThr287‐CaMKIIδ and pThr17‐PLN in comparison with I/R group treated with either intervention, recovery of heart function in this group was comparable. It can also be pointed out that CaMKII activity exhibits a bimodal pattern under I/R conditions.^19^, ^52^, ^53^ Thus, its role at earlier phases of reperfusion (after ischaemia) may be different to that of the later phases of reperfusion. It is therefore probable that CaMKII activity this late into reperfusion (as shown in our study) may actually be protective with regard to recovery of contractile function while it might no longer be coupled to signalling of programmed cell death, including necroptosis. In line with CaMKII signalling, we also looked at cMyBP‐C, which serves as a regulatory and structural protein of the sarcomere. Upon phosphorylation by various protein kinases, of which CaMKII plays an important role by the activation of Ser282 serving as a likely prerequisite for the activation of other sites,^54^ it accelerates cross‐bridge kinetics thereby enhancing both contraction and relaxation. In I/R and failing hearts, phosphorylation of this protein has been found to be lower and suggested as a significant marker of injury.^38^, ^55^ Interestingly, PC has not prevented I/R‐induced decrease in pSer282‐cMyBP‐C. On the other hand, Nec‐1s has normalized the phosphorylation state of pSer282‐cMyBP‐C in both I/R and PC hearts. Likewise, it increased the levels of pSer282‐cMyBP‐C besides pThr287CaMKII and pThr17‐PLN, in nonischaemic hearts. Thus, our data suggest that Nec‐1s can also affect pathways other than RIP1‐RIP3‐MLKL or elicit these effects indirectly. In support, as indicated earlier, CaMKII has been proposed to be a downstream of RIP3.^13^, ^47^ Thus, inhibition of RIP1 by Nec‐1s could result in a compensatory feedback as evidenced by elevated total RIP1 levels and increased pSer229‐RIP3 in perfused‐only hearts. Alternatively, some of these observations might be because of off‐target effects of Nec‐1s inhibition; however, we purposely used a relatively low (submicromolar) concentration of this inhibitor to minimize this possibility.^24^, ^56^

In addition to CaMKII‐linked pathway, antinecroptotic effects of PC were also investigated in regard of oxidative damage which in turn was found to be linked with necroptosis induction and execution.^57^ In spite of the mitigation of necroptosis evidenced by the lower levels of MLKL in the plasma membrane, the levels of TBARS were found to be unchanged in the PC group. The same is true for Nec‐1s ischaemic groups subjected or not to PC protocol. On the other hand, this substance showed a tendency to increase lipid peroxidation in nonischaemic hearts by unknown mechanisms. Thus, it seems that in our model, PC elicited cardioprotection because of necroptosis modulation which was, however, not associated with a decrease in oxidative damage of lipids. No action of PC on TBARS levels is in accordance with some previous studies.^58^, ^59^ On the other hand, other studies have reported a lower oxidative stress because of PC,^60^ a discrepancy that could be explained by different PC protocols.

Taken together, this study has shown that PC might be cardioprotective by providing antinecroptotic effects because of inhibiting MLKL translocation within the plasma membrane and that necroptosis inhibitor did not intensify its effect. Improvement of heart function in the preconditioned hearts was also not increased by Nec‐1s although it itself prevented myocardial stunning. Modification of oxidative stress is unlikely to be linked with these effects. Likewise, CaMKII‐mediated pathway seems to explain the effects of PC on ECC rather than on necroptosis. Thus, which particular mechanisms underlie the observed findings is an appealing future study goal.

## CONFLICT OF INTEREST

There are no conflicts of interest.

## AUTHOR'S CONTRIBUTIONS

AS, VFL, ML, MM and SČ performed the experiments; AS, VFL, SČ and TR analysed the data; AS, AA and TR contributed to the acquisition, analysis and interpretation of data; AA, AS and TR developed the concept; AS, ML and AA wrote the manuscript. All authors gave final approval.
