# Supplementary material for: Cardioprotection of ischaemic preconditioning is associated with inhibition of translocation of MLKL within the plasma membrane
Source: J Cell Mol Med. 2018 Jun 19;22(9):4183–96. doi: 10.1111/jcmm.13697 (PMC6111849; doi:10.1111/jcmm.13697)
Supplement: Supplementary file 1 [file JCMM-22-4183-s001.docx]

**Supplementary material for “Cardioprotection of ischemic preconditioning is associated with inhibition of translocation of MLKL within the plasma membrane and is not intensified by pharmacological inhibition of necroptosis”**

**Supplementary methods**

**Details of subcellular fractionation**

After initial homogenization of ventricular tissue in base buffer (100 mmol.l^-1^ imidazole-HCl pH=7.4, 20 mmol.l^-1^ 6-aminohexanoic acid, 500 mmol.l^-1^ sucrose, 5 mmol.l^-1^ MgCl_2_,) and a 30 min incubation on ice, samples were centrifuged at 800g for 20 minutes. After centrifugation, the supernatant was removed and used for the subsequent isolation of cytosolic and membrane fractions. The pellet was processed to isolate the nuclear fraction.

*Isolation of nuclear fraction:*

The pellet was resuspended in base buffer and was centrifuged at 500g for 15 minutes. Afterwards, the same step was repeated but at 1000g. Then the pellet was resuspended in base buffer and layered on top of a sucrose solution (2.1 mol.l^-1^ sucrose in base buffer) and centrifuged at 10000g for 30 minutes. The supernatant was then carefully removed, pellet was washed with base buffer, resuspended in base buffer supplemented with 1% (v/v) Triton X-100, mixed thoroughly for 20 minutes at 4°C and finally centrifuged at 1500g for 10 minutes. After discarding the supernatant, the pellet was washed with base buffer and solubilized in S buffer (base buffer with 1% v/v Triton X-100, 0.25% w/v Na-deoxycholate, 1% w/v SDS, 1 mmol.l^-1^ EDTA, 1 mmol.l^-1^ EGTA) with 10 U.ml^-1^ Benzonase (DE, Millipore). Sample was mixed for 10 minutes, centrifuged at 10000g and the final supernatant containing nuclear fraction was stored at -80°C.

*Isolation of membrane and cytoplasmic fractions:*

Stored supernatant from the first centrifugation step was centrifuged at 800g for 20 minutes and then was transferred to another tube, which was subsequently centrifuged at 10000g for 10 minutes. The pellet containing mostly mitochondria was discarded and 1 mol.l^-1^ CaCl_2_ was added to the supernatant (final concentration 10 mmol.l^-1^) to induce membrane vesicle aggregation, incubated on ice for 10 minutes and centrifuged at 18000g for 30 minutes. Supernatant containing the cytoplasmic fraction was transferred into another tube. The pellet was washed with base buffer, resuspended in S buffer and mixed for 15 minutes. Afterwards, SDS, EDTA and EGTA was added to reach the final concentration of 2% (w/v), 5 mmol.l^-1^ and 5 mmol.l^-1^, respectively. The solution was strongly mixed for 45 minutes to completely dissolve and stored at -80°C. Finally, the cytoplasmic fraction, which was set aside, had detergents and chelators added (final concentrations as in S buffer) and stored at -80°C.

**Antibodies used for immunoblotting**

Primary antibodies: rabbit anti-RIP1 (1:1000, SAB3500420, Sigma-Aldrich, USA), rabbit anti-RIP3 [rodent specific] (1:1000, #14401, Cell Signaling Technology, USA), rabbit anti-pSer232-RIP3 (1:1000, ab195117, Abcam, UK), rat anti-MLKL (1:500, MABC604, Millipore, USA), anti-PARP1 (1:1000, #9532, Cell Signaling Technology, USA), anti-p25-PARP1 (1:500, ab32064, Abcam, UK), rabbit anti-Bcl2 (1:1000, SAB3500420, Sigma-Aldrich, USA), rabbit anti-Bax (1:1000, #2772, Cell Signaling Technology, USA), rabbit anti-caspase-8 (1:1000, 04-573, Millipore, US), rabbit anti-Cleaved caspase-3 [5A1E] (1:500, #9664, Cell Signaling Technology, USA), rabbit anti-caspase-7 (1:500, #12827, Cell Signaling Technology, USA), rabbit anti-CaMKIIδ (1:2000, 15443-1-AP, Proteintech, USA), rabbit anti-pThr286-CaMKII (1:2000, #12716, Cell Signaling Technology, USA), mouse anti-PLN (1:3000, A010-14, Badrilla, UK), rabbit anti-pThr17-PLN (1:2000, A010-13AP, Badrilla, UK), mouse anti-cMYBP-C (1:5000, sc-137237, Santa Cruz Biotechnology, USA), rabbit anti-pSer282-cMYBP-C (1:2000, ALX-215-057, Enzo Life Sciences, USA), rabbit anti-H3 (1:3000, #4499, Cell Signaling Technology, USA), mouse anti-GAPDH (1:100000, 60004, Proteintech, USA), rabbit anti-Na/K-ATPase (1:500, #3010, Cell Signaling Technology, USA).

Secondary antibodies: donkey anti-rabbit IgG-HRP (1:50000, 711-035-152, Jackson Immunoresearch, USA), donkey anti-mouse IgG-HRP (1:50000, 715-065-150, Jackson Immunoresearch, USA), mouse anti-rat light chain specific IgG-HRP (1:50000, 112-035-175, Jackson Immunoresearch, USA).

**Supp. Fig. 1**

**Supplementary Figure 1**

Analysis of effects of IPC and Nec-1s on whole tissue oxidative stress in I/R-damaged hearts measured as TBARS in perfused-only (C) and treated hearts with vehicle (IR), IPC (PC), Nec-1s (IR+N) and their combination (PC+N). Data are presented as mean ± SEM, n = 6 / group. *p < 0.05.

**A**

**C**

**N**

**C**

**N**


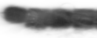

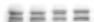

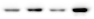


pSer^282^-cMYBP-C

cMYBP-C

PLN


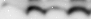


pThr^17^-PLN


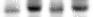

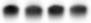


CaMKIIδ

pThr^287^-CaMKIIδ

csp-8

procsp-8


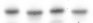


procsp-7


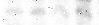


csp-7


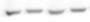

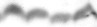


procsp-3

csp-3


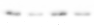

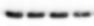


Bcl-2

Bax

PARP1


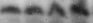


PARP1 (p25)


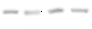

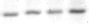

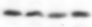


RIP3

RIP1

MLKL


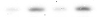


pSer^229^-RIP3


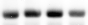

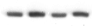

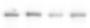


**C**

**N**

**C**

**N**

**C**

**N**

**C**

**N**

**C**

**N**

**C**

**N**

**RIP1**

**RIP3**

**pSer^229^**

**-RIP3/RIP3**

**ratio**

**MLKL**

**csp-8/**

**procsp-8**

**ratio**

**csp-3/**

**procsp-3**

**ratio**

**csp-7/**

**procsp-7**

**ratio**

**PARP1**

**PARP1**

**(p25)**

**Bcl-2/Bax**

**ratio**

**pThr^287^-**

**CaMKIIδ/**

**CaMKIIδ ratio**

**pSer^282^-**

**cMYBP-C/**

**cMYBP-C ratio**

**pThr^17^-PLN/**

**PLN ratio**

**B**

**C**

**D**

**E**

*****

*****

*****

*****

*****

*****

**F**

**p=0.062**


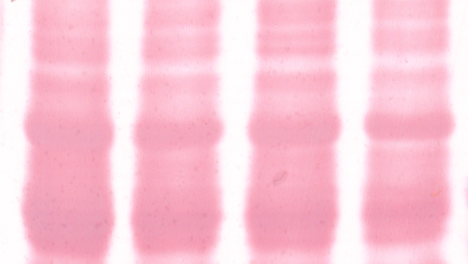


*Tot. prot.*

*Tot. prot.*

*Tot. prot.*

*Tot. prot.*


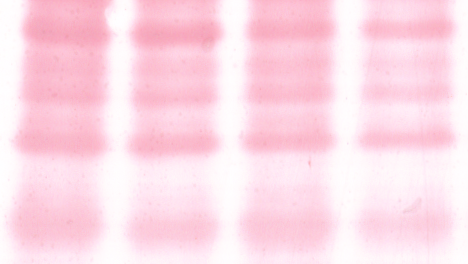

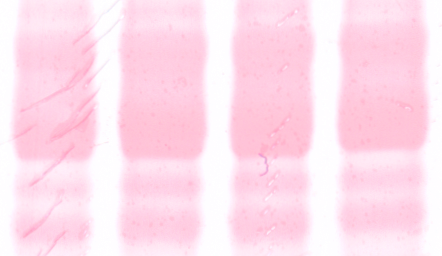

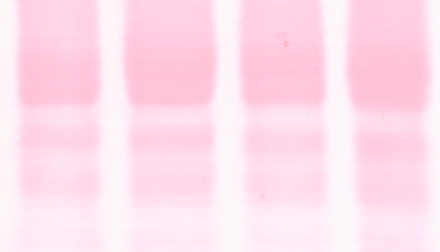


**Supp. Fig. 2**

**Supplementary Figure 2**

Analysis of effects of Nec-1s in non-ischemic hearts. **a**) Representative immunoblots and **b-e**) quantification of necroptotic (RIP1, RIP3, pSer229-RIP3/RIP3 ratio, MLKL), apoptotic (ratios of csp-8/csp-3/csp-7 to their respective proforms, PARP1, cleaved p25 PARP1, Bcl-2/Bax ratio), CaMKII-related pathways (pThr287-CaMKIIδ/CaMKIIδ ratio, pThr17-PLN/PLN ratio and pSer282-cMyBP-C/cMyBP-C ratio) and oxidative stress assessed by TBARS levels in perfused-only (C) and Nec-1s-perfused (N) hearts. Data are presented as mean ± SEM, n = 6 and 3 / group respectively. *p < 0.05.
